# Abrogation of IL-6-mediated JAK signalling by the cyclopentenone prostaglandin 15d-PGJ_2_ in oral squamous carcinoma cells

**DOI:** 10.1038/sj.bjc.6602055

**Published:** 2004-08-17

**Authors:** H Siavash, N G Nikitakis, J J Sauk

**Affiliations:** 1 Department of Diagnostic Sciences and Pathology, University of Maryland, Baltimore, MD 21201, USA; 2 Department of Biomedical Sciences, University of Maryland, Baltimore, MD 21201, USA; 3 Greenebaum Cancer Center, University of Maryland, Baltimore, MD 21201, USA

**Keywords:** 15d-PGJ_2_, IL-6, JAK, Stat3, oral squamous cell carcinoma

## Abstract

Cyclopentenone 15-deoxy-Δ^12,14^-prostaglandin J_2_ (15d-PGJ_2_) exerts antineoplastic effects on various types of human cancer. We recently showed that treatment with 15d-PGJ_2_ induces apoptosis accompanied by downregulation of the oncogenic signal transducer and activator of transcription 3 (Stat3) signalling in human oral squamous cell carcinoma (SCC) cells. The current study examines the effects of 15d-PGJ_2_ on the epidermal growth factor receptor (EGFR) and Janus Kinase (JAK)-mediated signalling pathways. Inhibition of Stat3 by 15d-PGJ_2_ was abolished by exogenous stimulation with transforming growth factor alpha (TGF-*α*), but not interleukin 6 (IL-6), supporting a selective effect of 15d-PGJ_2_ on IL-6-mediated signalling. Importantly, 15d-PGJ_2_ selectively abrogated constitutive and IL-6-mediated JAK phosphorylation without affecting EGFR-activated levels. Moreover, the inhibitory effect of 15d-PGJ_2_ on JAK signalling required the reactive *α*,*β*-unsaturated carbon within the cyclopentenone ring. Targeting of JAK signalling using a specific JAK inhibitor also abolished Stat3 phosphorylation and resulted in apoptosis in oral SCC cells. Our findings provide the first evidence for 15d-PGJ_2_–mediated downregulation of constitutive and IL-6-induced JAK signalling in cancer and support that JAK inhibition and suppression of EGFR-independent Stat3 activation by 15d-PGJ_2_ represent a promising approach for induction of apoptosis in oral SCC cells.

Signal transducer and activator of transcription (STAT) proteins are latent cytoplasmic transcription factors that typically become activated in response to extracellular signals such as growth factors and cytokines (Darnell Jr, 1997; Bromberg and Darnell Jr, 2000). Upon binding to their corresponding cell membrane receptors, cytokines and growth factors induce STAT tyrosine phosphorylation, mediated by cytokine receptor-associated tyrosine kinases (i.e. Janus Kinase family members, including Jak1, Jak2, Jak3 and Tyk2) and growth factor receptors with intrinsic kinase activity (e.g. epidermal growth factor receptor, EGFR), respectively (Darnell Jr, 1997; Bromberg and Darnell Jr, 2000). In addition, the Src family of nonreceptor tyrosine kinases has also been shown to phosphorylate STAT proteins (Bromberg and Darnell Jr, 2000). Activated STAT molecules form homo- or heterodimers and translocate to the nucleus, where they bind to the promoter region of specific target genes, thus regulating their transcription (Darnell Jr, 1997; Bromberg and Darnell Jr, 2000).

STATs have been identified as critical regulators of various normal cellular processes (Darnell Jr, 1997; Levy, 1999). However, persistent STAT activation has been convincingly implicated in oncogenesis (Bowman *et al*, 2000; Bromberg and Darnell Jr, 2000; Bromberg, 2001, 2002). It has been established that abnormal activation of STAT molecules (especially involving Stat3 and Stat5) stimulates cell proliferation and prevents apoptosis in a number of human tumours, including leukaemia, multiple myeloma, breast, prostate and non-small-cell lung cancer (Bromberg, 2002). Therefore, disruption of aberrant STAT activation in tumours, which typically depends on deregulation of specific upstream tyrosine kinases, has been proposed as a valid molecular target for cancer therapy (Bowman *et al*, 2000; Turkson and Jove, 2000; Bromberg, 2001, 2002; Buettner *et al*, 2002).

In head and neck squamous cell carcinoma (SCC), there is evidence that Stat3 constitutive activation is linked to cancer development and growth (Grandis *et al*, 2000; Song and Grandis, 2000; Kijima *et al*, 2002). Importantly, targeting of Stat3, through transfection of dominant-negative constructs or application of antisense oligonucleotide treatment, results in significant growth inhibition (Grandis *et al*, 1998; Song and Grandis, 2000). Furthermore, Stat3 antisense gene therapy leads to increased tumour apoptosis *in vivo*, which is associated with decreased Bcl-X_L_ protein expression (Grandis *et al*, 2000; Song and Grandis, 2000; Kijima *et al*, 2002).

Cyclopentenone prostaglandins, especially 15-deoxy-Δ^12,14^-prostaglandin J_2_ (15d-PGJ_2_), have been shown to exert antineoplastic effects on various types of human cancer (Kim *et al*, 1993; Ahn *et al*, 1998; Clay *et al*, 1999; Keelan *et al*, 1999; Butler *et al*, 2000; Chang and Szabo, 2000; Clay *et al*, 2001; Straus and Glass, 2001). These effects, frequently attributed to activation of peroxisome proliferator-activated receptor gamma (PPAR*γ*), have been recently proven to be at least partially mediated through PPAR*γ*-independent pathways (Clay *et al*, 2001, 2002; Straus and Glass, 2001; Hsiang and Straus, 2002; Liu *et al*, 2003). Similarly, we recently demonstrated that 15d-PGJ_2_ inhibits cell growth and induces apoptosis in oral SCC utilising PPAR*γ*-independent mechanisms (Nikitakis *et al*, 2002). Moreover, we suggested that the effects of 15d-PGJ_2_ on oral SCC cells may be related to its ability to downregulate Stat3 (Nikitakis *et al*, 2002). In the present study, we investigated the effect of 15d-PGJ_2_ on tyrosine kinases that regulate growth and survival of oral SCC cells, including JAKs and EGFR. Our results indicate that 15d-PGJ_2_ targets JAK signalling independent of EGFR signalling and suggest that inhibition of IL-6-mediated JAK signalling and suppression of EGFR-independent Stat3 activation may represent a novel therapeutic approach in oral cancer.

## MATERIALS AND METHODS

### Cell lines and cell culture

All experiments were performed using established human oral SCC cell lines (SCC-4, -9, -15 and -25) obtained from American Type Culture Collection (ATCC) (Manassas, VA, USA). Cells were cultured in a 1 : 1 mixture of Ham's F12 and Dulbecco's modified Eagle's medium (DMEM) containing 10% foetal bovine serum (FBS), 100 U of penicillin, 100 *μ*g ml^−1^ streptomycin and 0.4 *μ*g ml^−1^ hydrocortisone (Sigma Chemical Co., St Louis, MO, USA) at 37°C in a 5% CO_2_ air atmosphere. Cells were subcultured by disaggregation with trypsin (0.1%)-EDTA (0.01%) in phosphate-buffered saline (PBS) at pH 7.5.

### Western blot analysis

Cells were plated in six-well plates at 5x10^4^ cells well^−1^ and were allowed to grow to 80% confluency. A measure of 10, 20 or 40 *μ*M 15d-PGJ_2_ (Cayman Chemical, Ann Arbor, MI, USA) dissolved in 100% DMSO, 50 *μ*M AG490 (Calbiochem, San Diego, CA, USA) dissolved in 100% DMSO, 100 nM PD153035 (Calbiochem), 20 or 40 *μ*M 9,10-dihydro-15-Deoxy-Δ^12,14^-PGJ_2_ (CAY10410) (Cayman Chemical) dissolved in 100% DMSO, 20 ng ml^−1^ rIL-6 (Calbiochem) or 25 ng ml^−1^ TGF-*α* (Calbiochem) was added to normal medium (NM). The final concentration of DMSO did not exceed 0.1%. Alternatively, cells were pretreated with 15d-PGJ_2_ for 1 h and subsequently treated with rIL-6 or TGF-*α*. Following incubation for various time periods, the cells were washed twice with cold PBS, lysed in RIPA buffer (50 mM Tris (pH 7.4), 150 mM NaCl, 1% Triton X-100, 1% deoxycholic acid, sodium salt, 0.1% sodium dodecyl sulphate (SDS), 100 *μ*g ml^−1^ phenylmethylsulphonyl fluoride, 1 *μ*g ml^−1^ aprotinin, 1 mM dithiothreitol and 1 mM sodium orthovanadate) for 10 min, and scraped. The extracts were centrifuged at 40 000 **g** for 15 min at 4°C. Protein concentrations were measured and equalised using Bio-Rad protein assay (Bio-Rad Laboratories, Richmond, CA, USA) according to the manufacturer's instructions.

Western blot analysis was performed using phospho-Stat3 (Tyr705) antibody (1 : 500) (Cell Signaling Technology, Beverly, MA, USA), phospho-Jak2 (Tyr1007, Tyr1008) antibody (0.5 *μ*g ml^−1^) (Upstate Biotechnology, Lake Placid, NY, USA) or SOCS3 (M-20) antibody (1 : 200) (Santa Cruz Biotechnology, Santa Cruz, CA, USA) according to the manufacturer's instructions. Blots were stripped (20 mM dithiothreitol, 2% SDS and 67.5 mM Tris-HCl (pH 6.7)) and then reprobed with Stat3 antibody (1 : 1000) (Cell Signaling Technology), Jak2 antibody (1 : 1000) (Upstate Biotechnology) or *β*-actin antibody (Sigma-Aldrich, St Louis, MO, USA), respectively.

### Immunoprecipitation

Cells (5 × 10^4^ well^−1^) were allowed to grow to 80% confluency and rIL-6 was added to normal medium at 25 ng ml^−1^ concentration. Alternatively, cells were treated with 20 *μ*M 15d-PGJ_2_, 40 *μ*M CAY10410 or 50 *μ*M AG490, dissolved in 100% DMSO. Cells were also pretreated with 15d-PGJ_2_ and subsequently treated with rIL-6. The final concentration of DMSO did not exceed 0.1%. Following incubation for various time periods, the cells were lysed in RIPA buffer as described above. Immunoprecipitation of Jak1, Jak2, EGFR and SHP2 were performed using 1 mg of whole cell lysate. Jak1 antibody (4 *μ*g ml^−1^) (Upstate Biotechnology, Lake Placid, NY, USA), EGFR antibody (1 : 100) (Cell Signaling Technology) or SHP2 (5 *μ*g ml^−1^) (BD Biosciences Pharmingen, San Diego, CA, USA) were added to the precleared supernatant and incubated overnight at 4°C. Lysates were then incubated with protein A magnetic beads (New England Biolabs, Beverly, MA, USA) for 2 h. Beads were washed and resuspended in sample loading buffer (187.5 mM Tris-HCl (pH 6.8), 6% (w v^−1^) SDS, 30% glycerol, 150 mM DTT, 0.03% (w v^−1^) bromophenol blue and 2% *β*-mercaptoethanol). Western blot analysis was performed using phospho-tyrosine-specific monoclonal antibody 4G10 (1 *μ*g ml^−1^) (Upstate Biotechnology). Membranes were reprobed with Jak1 (1 : 1000), EGFR (1 : 1000) or SHP2 (1 : 2500) antibodies as controls.

### Cell growth inhibition

Cells were plated at 5 × 10^4^ cells well^−1^ in 24-well plates containing normal growth medium. After 24 h, DMSO at 0.1% or AG490 at 25, 50 or 100 *μ*M was added. The final concentration of DMSO did not exceed 0.1%. Following incubation for 24, 48 or 72 h, cells were enzymatically removed and counted using a Coulter Counter (Coulter Model ZI, Coulter Corporation, Miami, FL, USA). All analyses were performed in triplicate.

### Measurement of apoptosis

Cells were treated with either 0.1%. DMSO or AG490 at 50 or 100 *μ*m for 48 h. Cells were washed twice with cold PBS and resuspended in 1 × binding buffer. Early apoptotic changes were identified by Annexin V-FITC and PI staining (BD Biosciences). The extent of apoptosis was assessed by relative fluorescence intensity using a FACScan and Cell Quest software (Becton Dickenson) as described (Nikitakis *et al*, 2002). All analyses were performed in duplicate.

### Statistical analysis

A two-way analysis of variance (ANOVA) test and Tukey test were used to assess the presence of statistically significant differences between groups (SigmaStat 3.0, SPSS Inc, Chicago, IL, USA); *P*⩽0.05 was considered statistically significant.

## RESULTS

### Inhibition of IL-6-mediated Stat3 phosphorylation by cyclopentenone prostaglandin 15d-PGJ_2._

**Figure 1:**
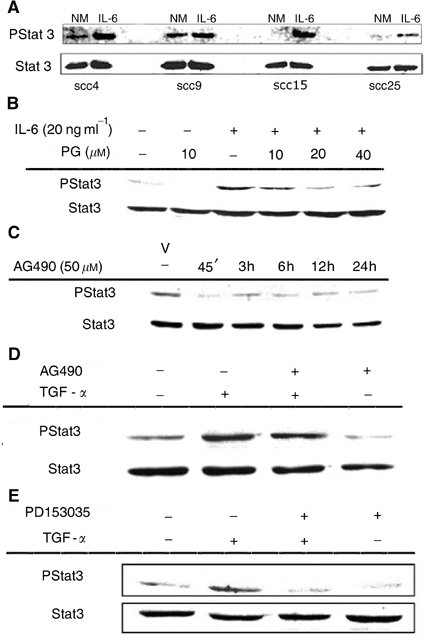
15d-PGJ_2_ inhibits IL-6-induced Stat3 phosphorylation in oral SCC cells. (**A**) Oral SCC cells were treated with normal medium (NM) or rIL-6 (20 ng ml^−1^) for 24 h. (**B**) Oral SCC25 cells were treated with NM, 15d-PGJ_2_ (PG 10 *μ*M) for 1 h or rIL-6 (20 ng ml^−1^) for 45 min. Alternatively, cells were treated rIL-6 (20 ng ml^−1^) for 45 min in the presence of 10, 20 or 40 *μ*M pretreatment with 15d-PGJ_2_. (**C**) AG490 inhibits Stat3 constitutive phosphorylation. Oral SCC9 cells were treated with either 0.1% DMSO as vehicle (V) or AG490 (50 *μ*M) for 45 min, 3, 6, 12 or 24 h. (**D** and **E**) Oral SCC9 cells were either untreated or supplemented with 25 ng ml^−1^ TGF-*α* for 45 min in the presence or absence of 1 h pretreatment with (**D**), AG490 (50 *μ*M) or (**E**) PD153035 (100 ng ml^−1^). Cell lysates were blotted with phospho-Stat3 antibody (Y705) and subsequently stripped and reprobed with Stat3 antibody. Comparable results were obtained from other cell lines used.

In our previous study, we reported that treatment with 15d-PGJ_2_ (20 *μ*M) suppresses the levels of constitutively phosphorylated Stat3 (Nikitakis *et al*, 2002). To assess if 15d-PGJ_2_ modulates upstream signalling leading to Stat3 repression, we tested the effect of 15d-PGJ_2_ on IL-6-mediated Stat3 phosphorylation. In the absence of pretreatment with 15d-PGJ_2_, IL-6 stimulation induced Stat3 phosphorylation (Figure 1A

). However, in the presence of 10 *μ*M 15d-PGJ_2_, IL-6-induced Stat3 tyrosine phosphorylation was attenuated as early as 45 min. Pretreatment with 20 or 40 *μ*M 15d-PGJ_2_ resulted in further reduction of IL-6-stimulated Stat3 phosphorylation (Figure 1B). This decrease in Stat3 tyrosine phosphorylation could not be attributed to a reduction in total cellular Stat3 levels, which remained stable despite the various treatments (or combinations thereof).

### 15d-PGJ_2_ abrogates the activation of JAKs

It is well established that members of the JAK family of kinases play an essential role in transducing signals from IL-6 type family of receptors to downstream effectors including STATs (Heinrich *et al*, 2003). In order to test the possibility that JAKs contribute to aberrant Stat3 activity in oral SCC cells, we examined the effect of AG490, a selective JAK inhibitor (Meydan *et al*, 1996; Nielsen *et al*, 1997), on Stat3 phosphorylation. AG490 (50 *μ*M) inhibited constitutive Stat3 phosphorylation during treatment for up to 24 h (Figure 1C). Moreover, treatment with the EGFR ligand, TGF-*α* (25 ng ml^−1^), reversed the AG490-mediated Stat3 repression (Figure 1D), while 100 nM treatment with a selective EGFR inhibitor, PD153035, abolished TGF-*α*-mediated Stat3 phosphorylation. (Figure 1E).

**Figure 2:**
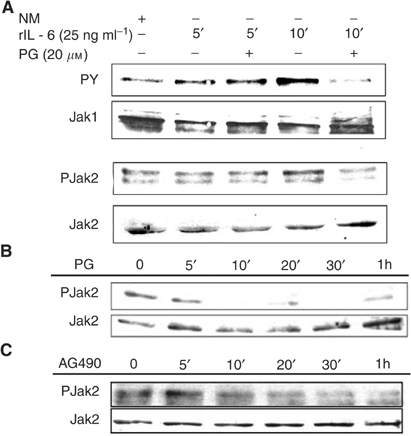
15d-PGJ_2_ abrogates JAK phosphorylation in oral SCC cells. (**A**) 15d-PGJ_2_ abrogates IL-6-induced JAK phosphorylation in oral SCC cells. Oral SCC25 cells were treated with NM, or rIL-6 (25 ng ml^−1^) for 5 or 10 min in the presence or absence of pretreatment with 15d-PGJ_2_ (20 *μ*M) for 30 min, followed by immunoprecipitation for Jak1 and Western blotting for phosphorylated (PY) and total Jak1. For Jak2, cell lysates were blotted with phospho-Jak2 (Y1007, Y1008) antibody and subsequently stripped and reprobed with Jak2 antibody. (**B**) 15d-PGJ_2_ inhibits constitutive JAK phosphorylation in oral SCC cells. Oral SCC9 cells were either untreated or treated with 15d-PGJ_2_ (20 *μ*M) for 5 min, 10 min, 20 min, 30 min and 1 h. (**C**) Effect of AG490 on constitutive JAK phosphorylation in oral SCC cells. Oral SCC9 cells were either untreated or treated with AG490 (50 *μ*M) for 5 min, 10 min, 20 min, 30 min and 1 h. In (**B**) and (**C**), cell lysates were blotted with phospho-Jak2 (Y1007, Y1008) antibody and subsequently stripped and reprobed with Jak2 antibody.

Given our finding that JAK inhibition can repress aberrant Stat3 signalling in oral SCC, we assessed the effect of 15d-PGJ_2_ on the induction of JAK phosphorylation. When SCC cells were pretreated with 15d-PGJ_2_ (20 *μ*M), a time-dependent reduction of IL-6-induced Jak1 phosphorylation was observed (Figure 2A

). Similarly, treatment with 15d-PGJ_2_ (20 *μ*M) abrogated Jak2 phosphorylation even in the presence of exogenous IL-6 for 10 min (Figure 2A). Total Jak1 and Jak2 protein levels remained unchanged following either treatment, indicating a specific effect of 15d-PGJ_2_ on JAK phosphorylation. In addition, treatment with 15d-PGJ_2_ (20 *μ*M) inhibited Jak2 constitutive phosphorylation after 10 min (Figure 2B). Similarly, AG490 (50 *μ*M) reduced constitutive Jak2 phosphorylation levels as early as 10 min of treatment (Figure 2C). Interestingly, treatment with AG490 for 10 min also partially inhibited constitutive Jak1 phosphorylation (Figure 4B).

### 15d-PGJ_2_ does not inhibit EGFR-mediated Stat3 signalling

**Figure 3:**
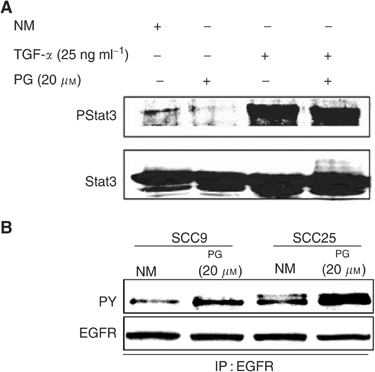
Effect of 15d-PGJ_2_ on EGFR-mediated Stat3 signalling. (**A**) Representative oral SCC cells (SCC9 and SCC25) were treated with NM, 15d-PGJ_2_ (20 *μ*M) for 45 min, or stimulated with TGF-*α* (25 ng ml^−1^) for 45 min in the presence or absence of 1 h pretreatment with 15d-PGJ_2_ PG (20 *μ*M). Cells were blotted with phospho-Stat3 antibody (Y705) and subsequently stripped and reprobed with Stat3 antibody. (**B**) Representative oral SCC cells (SCC9 and SCC25) were treated with NM or 15d-PGJ_2_ PG (20 *μ*M) for 10 min. EGFR constitutive phosphorylation was determined by immunoprecipitation with EGFR antibody and Western blotting for phosphorylated (PY) and total EGFR.

Given that autocrine and paracrine activation of the EGFR signalling pathway by TGF-*α* has been linked to Stat3 activation in head and neck SCC cells (Grandis *et al*, 2000; Song and Grandis, 2000), the possible involvement of EGFR activity in the inhibitory effect of 15d-PGJ_2_ on Stat3 was examined. To assess the effect of 15d-PGJ_2_ on EGFR-mediated activation of Stat3, oral SCC cells were stimulated with exogenous TGF-*α* in the presence or absence of 15d-PGJ_2_. Pretreatment with 15d-PGJ_2_ (20 *μ*M) failed to inhibit Stat3 phosphorylation in cells induced by exogenous TGF-*α* (Figure 3A

). Moreover, in the presence of 15d-PGJ_2_, downregulation of EGFR constitutive phosphorylation was not observed. On the contrary, a slight increase in EGFR constitutive phosphorylation was apparent following 15d-PGJ_2_ treatment (Figure 3B).

### Cyclopentenone structural requirement for the inactivation of JAK and Stat3 by 15d-PGJ_2_

**Figure 4:**
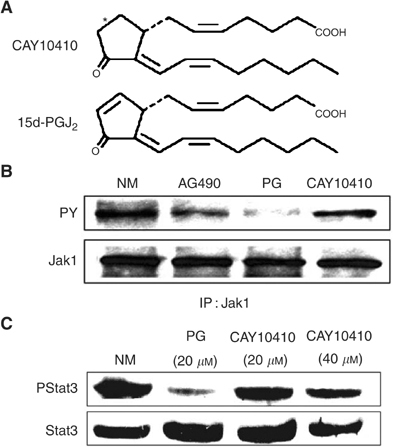
15d-PGJ_2_-mediated inhibition of JAK/Stat3 signalling requires the reactive cyclopentenone ring system. (**A**) Structure of 9,10-dihydro-15-Deoxy-Δ^12,14^-PGJ_2_ (CAY10410) and 15d-PGJ_2_. (**B**) 15d-PGJ_2_, but not CAY10410, abrogates constitutive JAK phosphorylation in oral SCC cells. Oral SCC9 cells were treated with NM, AG490 (50 *μ*M), 15d-PGJ_2_ (20 *μ*M) or CAY10410 (40 *μ*M) for 10 min. Cell lysates were immunoprecipitated with Jak1 antibody and Western blotted for phosphorylated (PY) and total Jak1. (**C**) Effect of CAY10410 on Stat3 phosphorylation. Oral SCC9 cells were treated with NM, 15d-PGJ_2_ (20 *μ*M) or CAY10410 (20 and 40 *μ*M) for 45 min. Cells were blotted with phospho-Stat3 antibody (Y705) and subsequently stripped and reprobed with Stat3 antibody.

Because PPAR*γ* activation is insufficient to drive the Stat3 inhibitory effects of 15d-PGJ_2_ in oral SCC cells (Nikitakis *et al*, 2002), we tested the possibility that specific downmodulation of JAK and Stat3 activation following treatment with 15d-PGJ_2_ requires the reactive electrophilic *α*,*β*-unsaturated carbonyl group within the cyclopentenone ring. This electrophilic carbon can react covalently with a number of nucleophiles, such as cysteine residues in cellular proteins, glutathione or sulphhydryls producing Michael addition-type reactions (Straus and Glass, 2001). Strikingly, treatment of oral SCC cells with 9,10-dihydro-15-Deoxy-Δ^12,14^-PGJ_2_ (CAY10410), a structurally modified 15d-PGJ_2_ that lacks the *α*,*β*-unsaturated carbonyl (Figure 4A

), failed to inhibit Jak1 (Figure 4B) and Stat3 (Figure 4C) constitutive phosphorylation levels. In contrast, in the presence of a reactive cyclopentenone ring system, 15d-PGJ_2_ maintained a potent inhibitory effect on JAK (Figure 4B) and Stat3 (Figure 4C) constitutive phosphorylation.

### AG490 induces growth inhibition and apoptosis in oral SCC cells

**Figure 5:**
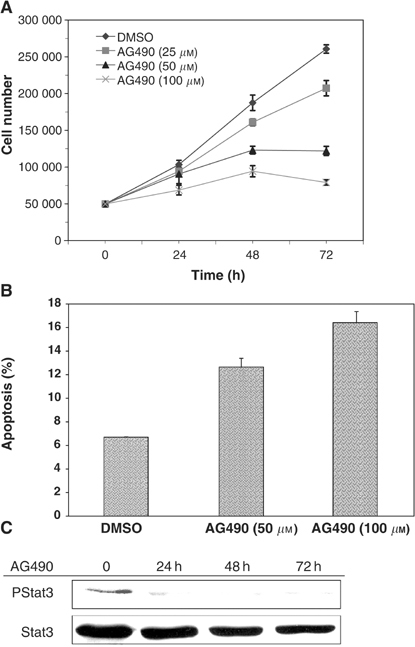
AG490 reduces cell growth and induces apoptosis in oral SCC cells. (**A**) Oral SCC9 and SCC25 cells were treated with vehicle (0.1% DMSO) or AG490 at 25, 50 and 100 *μ*M and cell growth was assessed following 24, 48 and 72 h of treatment using a Coulter Counter. Data are expressed as mean values±s.d. for representative cells. There was a significant growth inhibitory effect of AG490 (*P*⩽0.05) with respect to both dose and time using a two-way analysis of variance (ANOVA) test and Tukey test. (**B**) Representative oral SCC cells (SCC25) were treated with vehicle (0.1% DMSO) or AG490 at 50 *μ*M, and 100 *μ*M for 48 h. Annexin V-FITC assay revealed induction of apoptosis in cells treated with AG490 compared to vehicle. (**C**) Oral SCC9 cells were treated with vehicle (0.1% DMSO) or AG490 at 100 *μ*M and Stat3 constitutive phosphorylation was assessed following 24, 48 and 72 h of treatment. Cells were blotted with phospho-Stat3 antibody (Y705) and subsequently stripped and reprobed with Stat3 antibody.

In order to determine the physiologic consequence of JAK inhibition in oral SCC cells, cells were treated with a selective JAK inhibitor. Treatment with AG490 resulted in a significant (*P*⩽0.05) dose-dependent and time-dependent growth reduction in SCC cells (Figure 5A

). In addition, analysis of cells treated with AG490 by Annexin V-FITC and propidium iodide staining revealed the AG490-induced cell growth inhibition resulted in a dose-dependent increase in apoptosis (Figure 5B). Moreover, the antineoplastic effects of AG490 in oral SCC cells also correlated with the abrogation of Stat3 constitutive phosphorylation at 24, 48 and 72 h (Figure 5C).

### Effect of 15d-PGJ_2_ on negative regulators of Stat3 signalling

**Figure 6:**
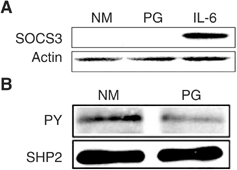
Effect of 15d-PGJ_2_ on negative regulators of Stat3 signalling. (**A**) Effect of 15d-PGJ_2_ on SOCS3 expression. SCC9 cells were either treated with 20 *μ*M, 15d-PGJ_2_ (PG) or rIL-6 (25 ng ml^−1^) for 1 h. Cells were blotted with SOCS3 (M-20) antibody and subsequently stripped and reprobed with actin control antibody. (**B**) Effect of 15d-PGJ_2_ on SHP2 phosphorylation. SCC9 cells were treated with 20 *μ*M, 15d-PGJ_2_ (PG) for 10 min. Cell lysates were immunoprecipitated with SHP2 antibody and Western blotted for phosphorylated (PY) and total SHP2.

Because negative regulators of STAT signalling, including suppressors of cytokine signalling (SOCS) and Src homology 2 domain-containing protein phosphatases (SHPs), may contribute to the 15d-PGJ_2_-mediated repression of JAK and Stat3 phosphorylation, the effect of 15d-PGJ_2_ on SOCS3 expression and SHP2 activity was examined. SOCS proteins are transcriptional targets of STATs and function in a classic negative feedback loop to inhibit further STAT activation by interacting with either JAK catalytic or receptor sites (Starr and Hilton, 1998, 1999). In this regard, treatment with IL-6 for 1 h potently induced SOCS3 protein levels in oral SCC cells (Figure 6A

). In contrast, treatment with 15d-PGJ_2_ (20 *μ*M) for 1 h did not have an effect on SOCS3 protein expression (Figure 6A). Moreover, we assessed the effect of 15d-PGJ_2_ on SHP2 phosphorylation. Treatment with 15d-PGJ_2_ (20 *μ*M) for 10 min did not upregulate SHP2 phosphorylation in oral SCC cells (Figure 6B).

## DISCUSSION

In an attempt to pinpoint the molecular target of 15d-PGJ_2_ in oral SCC, we examined the effect of 15d-PGJ_2_ on critical regulators of the Stat3 signalling complex, including the IL-6/JAK and the TGF-*α*/EGFR pathways. Induction of these two signalling pathways with exogenous ligands had opposite effects on 15d-PGJ_2_ activity in that only TGF-*α*, but not IL-6, stimulation could reverse 15d-PGJ_2_-induced Stat3 downregulation. Moreover, 15d-PGJ_2_ attenuated JAK phosphorylation in the presence or absence of IL-6 treatment, while modestly increased EGFR constitutively phosphorylated levels. It is noteworthy that the downmodulation of both Jak1 and Jak2 by 15d-PGJ_2_ preceded the inhibitory effects on IL-6-mediated Stat3 activation. Based on these observations, we suggest that the preferential target of 15d-PGJ_2_ is the IL-6/JAK signalling pathway, of which downmodulation results in the inhibition of Stat3 activation and induction of apoptosis.

Stat3 constitutive activation has been previously linked to aberrant TGF-*α*/EGFR autocrine or paracrine stimulation in head and neck SCC (Grandis *et al*, 2000; Song and Grandis, 2000; Leong *et al*, 2002). However, recent evidence points to the contribution of Src in EGFR-mediated Stat3 activation (Xi *et al*, 2003) and supports the existence of EGFR-independent Stat3 oncogenic properties (Kijima *et al*, 2002). Moreover, head and neck and oral SCC cells express a number of proinflammatory and proangiogenic cytokines, including IL-6, which may participate in Stat3 activation through binding to cytokine receptors and activation of receptor-associated JAK molecules (Chen *et al*, 1998, 1999; Ondrey *et al*, 1999). The recent observation that IL-6 stimulation of the gp130 results in constitutive activation of Stat3 independently of EGFR in head and neck SCC cells is also of particular importance (Sriuranpong *et al*, 2003). In this regard, we suggest that JAKs may serve a novel molecular target in oral SCC cells. This hypothesis is further corroborated by our findings that targeting of JAK activity by the specific kinase inhibitor AG490 abrogates constitutive Stat3 activation and induces growth inhibition accompanied by apoptotic cell death in oral SCC cells.

In that JAKs play an essential role in driving oncogenic Stat3 signalling (De Vos *et al*, 2000; Ni *et al*, 2000; Zhang *et al*, 2000; Li and Shaw, 2002; Rane and Reddy, 2002), the ability of 15d-PGJ_2_ to downmodulate IL-6/JAK signalling may also in part, explain the antineoplastic properties of this agent against other types of human cancer. For example, 15d-PGJ_2_ has been shown to have potent PPAR*γ*-independent proapoptotic effects on breast cancer cells (Clay *et al*, 2001, 2002), which exhibit constitutive Stat3 activation linked to cooperative activity of Src and JAK family tyrosine kinases (Garcia *et al*, 2001).

Further investigations should focus on elucidating the mechanisms by which 15d-PGJ_2_ inhibits JAK. The fact that exogenous stimulation with IL-6 was unable to reverse 15d-PGJ_2_-mediated JAK inhibition suggests that the target of 15d-PGJ_2_ effects is the kinase, but not the ligand. Consistent with this view, we have not observed downregulation of IL-6 levels after treatment with 15d-PGJ_2_ (unpublished data). Instead, JAK inhibition may be the result of direct interaction between 15d-PGJ_2_ and JAK, especially considering the ability of 15d-PGJ_2 _molecules to bind to and modify specific proteins through their reactive *α*,*β*-unsaturated carbonyl group within the cyclopentenone ring (Straus and Glass, 2001). In view of the latter, our data highlighting the requirement of the reactive cyclopentenone ring system for 15d-PGJ_2_-mediated repression of JAK and Stat3 constitutive phosphorylation are of particular importance. Moreover, 15d-PGJ_2_ possesses the ability to induce reactive oxygen species, which have been shown to contribute to the biological activities of cyclopentenone prostaglandins, including cytotoxic effects of 15d-PGJ_2_ (Kondo *et al*, 2001; Li *et al*, 2001; Lennon *et al*, 2002). The possible involvement of redox-sensitive mechanisms in the function of 15d-PGJ_2_ as an antineoplastic agent that induces cell growth inhibition and IL-6/JAK/Stat3 suppression in oral SCC cells is currently under investigation.

Alternatively, 15d-PGJ_2_, directly or through production of other mediators, may induce negative regulators of JAK activity, such as SOCS or SHP2. In this respect, stimulation with 15d-PGJ_2_ has been shown to induce the transcription of SOCS1 and SOCS3 and to activate SHP2, which in turn suppress inflammatory interferon signalling mediated by the JAK/STAT pathway in primary astrocytes (Park *et al*, 2003). However, we did not observe upregulation of SOCS3 and SHP2 during 15d-PGJ_2_-mediated repression of JAK phosphorylation in oral SCC cells. Although our findings suggest that the inhibition of JAK/STAT signalling by 15d-PGJ_2_ may require other mechanisms in oral SCC cells, the possible involvement of other negative regulators of JAK activity (Starr and Hilton, 1998; Starr and Hilton, 1999) warrants further investigation.

Negative regulation of Stat3 has also been associated with activation of mitogen-activated protein kinases (MAPKs), including ERKs, JNKs and p38 MAPK, possibly involving suppression of JAK activity (Sengupta *et al*, 1998; Bode *et al*, 1999; Lim and Cao, 1999; Ahmed and Ivashkiv, 2000; Bode *et al*, 2001). These observations, along with recent findings supporting activation of MAPKs by 15d-PGJ_2_ in a number of cells (Wilmer *et al*, 2001; Lennon *et al*, 2002), provide another possible explanation for 15d-PGJ_2_ effects on Stat3 signalling.

Although the mechanisms by which 15d-PGJ_2_ causes suppression of the IL-6-mediated JAK signalling in oral SCC necessitate further investigation, our findings provide a novel molecular explanation for the antineoplastic properties of cyclopentenone prostaglandins, which may facilitate their optimal use as therapeutic agents.
